# Unnerving Cough in CANVAS: Cough Hypersensitivity Despite Airway Nerve Depletion

**DOI:** 10.1007/s00408-025-00838-y

**Published:** 2025-08-08

**Authors:** Barnaby Hirons, Katherine Rhatigan, William McNulty, Richard D. Turner, James H. Hull, Caroline J. Jolley, Robert D. Hadden, Ana Ribeiro, Andrea Cortese, Peter S. P. Cho, Safa Al-Sarraj, Jordi Serra, Peter Bannister, Chadwick B. Smith, Matthew G. Drake, Surinder S. Birring

**Affiliations:** 1 Faculty of Life Sciences & Medicine, Centre for Human and Applied Physiological Sciences, King’s College London, London, UK; 2 Department of Respiratory Medicine, King’s College Hospital, London, UK; 3 School of Medicine and Dentistry, Griffith University, Southport, QLD Australia; 4 Department of Respiratory Medicine, Princess Alexandra Hospital, Brisbane, QLD Australia; 5 Department of Respiratory Medicine, Royal Brompton Hospital, London, UK; 6 Department of Neurology, King’s College Hospital, London, UK; 7 Department of Clinical Neurophysiology, King’s College Hospital, London, UK; 8 Department of Neuromuscular Disease, UCL Queen Square Institute of Neurology, London, UK; 9 Department of Brain and Behaviour Sciences, University of Pavia, Pavia, Italy; 10 Department of Neuropathology, King’s College Hospital, London, UK; 11 Division of Pulmonary, Allergy, and Critical Care Medicine, Oregon Health & Science University, Portland, OR USA; 12 Department of Respiratory Medicine, Chest Unit, Cheyne Wing, King’s College Hospital, Denmark Hill, London, SE5 9RS UK

**Keywords:** CANVAS, Cough hypersensitivity, Lung biopsy, Airway innervation

## Abstract

**Introduction:**

Cerebellar ataxia with neuropathy and vestibular areflexia syndrome (CANVAS) is a genetic neurodegenerative condition associated with chronic cough and cough hypersensitivity. The neuropathic mechanisms underlying cough in CANVAS are unknown. In a father and son with CANVAS-associated cough, we investigated clinical and neuropathophysiological features including bronchial and skin biopsies.

**Methods:**

Patients completed assessments for cough severity (visual analogue scale, VAS), impact (Leicester Cough Questionnaire, LCQ), triggers (Cough Hypersensitivity Questionnaire), objective frequency with Leicester Cough Monitor, and reflex sensitivity with capsaicin cough challenge. Bronchoscopic airway biopsies were analysed for nerve morphology and compared to a healthy control. Neurological assessments included skin biopsies, nerve conduction studies, and microneurography.

**Results:**

The father (age 62) and son (age 37) had advanced and early CANVAS, with a refractory chronic cough of 37 and 9 years duration, respectively. The cough in the father and son was of moderate severity (VAS 58 and 54 mm) and impact (LCQ score 15.9 and 13.1), with raised objective cough frequencies of 6 and 16 coughs hr^−1^, and heightened cough reflex sensitivity to capsaicin with concentrations to evoke five coughs (C5) of 14.9 and 3.3 μmol L^−1^, respectively. Bronchoscopic airway biopsies demonstrated severely depleted sensory small nerve fibres in the father and son compared to a healthy control: median (IQR) total nerve length 0 (0–0) and 0 (0–125) μm vs 944 (461–1323) μm, respectively. Skin biopsies showed absent intraepidermal nerve fibres, with densities of 0.0 fibres.mm^−1^ in both patients. Functional microneurography revealed nociceptor fibre paucity and dysfunction.

**Conclusion:**

In CANVAS, despite the loss of bronchial and cutaneous nerve fibres, there was heightened cough reflex sensitivity. Further studies are needed to elucidate underlying neural mechanisms.

## Introduction

Cerebellar ataxia with neuropathy and vestibular areflexia syndrome (CANVAS) is an autosomal recessive neurodegenerative condition that manifests as progressive sensory loss and gait imbalance [1]. In 2019, the genetic cause for CANVAS was discovered: biallelic repeat intronic expansions in AAGGG in intron 2 of replication factor C subunit 1 (*RFC1*) gene [1]. CANVAS is associated with depletion of large and small sensory nerve fibres (including unmyelinated C-fibres) [1, 2]. Chronic cough (cough lasting > 8 weeks) is a common symptom of CANVAS (64% of patients) and often precedes the onset of other neurologic findings by years or even decades [1]. CANVAS-associated cough is frequently refractory to treatment of possible contributing causes (e.g. asthma, gastroesophageal reflux [GORD], and rhinosinusitis), impactful, and has features of cough hypersensitivity with multiple cough triggers [3–5]. Furthermore, CANVAS cough is associated with cough reflex hypersensitivity to capsaicin, comparable to refractory chronic cough (RCC) without CANVAS [3].

The mechanism of cough in CANVAS remains unclear [4]. In RCC there is evidence of increased bronchial innervation with abnormal neural branching [6]. In this report we describe detailed neuropathophysiology of cough in two cases of CANVAS, at early and late presentation, utilising anatomical and functional investigations and compared to healthy donors.

## Methods

### Patient Characteristics

We describe a father and son, both diagnosed with refractory chronic cough (RCC) and CANVAS. RCC was defined as chronic cough that persists despite guideline-directed investigation and management of contributing causes [7]. CANVAS was established based on compatible clinical features and biallelic intronic AAGGG repeat RFC1 expansions on gene sequencing. As CANVAS has autosomal recessive inheritance, the mother of the son was tested and confirmed to be an RFC1 carrier (monoallelic RFC1 repeat expansions), consistent with the pseudodominant inheritance pattern observed in this family. Both the father and son had never smoked, and no other causes of cough were identified (Table 1). The patients provided written informed consent for procedures and case-series publication. A healthy lung was obtained from a 29-year-old Caucasian male post-mortem donor through the Pacific Northwest Transplant Bank (Cascade Alliance).

**Table 1:** Demographics and cough characteristics in CANVAS-associated chronic cough

	Father	Son
Demographics		
Age (years)	62	37
BMI (kg.m^−2^)	21	22
Medication	Pregabalin, minimal self-reported benefit to cough	Nortriptyline, minimal self-reported benefit to cough
Cough characteristics		
Duration of cough	37 years	9 years
Cough severity VAS (0–100)	58 mm	54 mm
LCQ total score (3–21)	15.9	13.1
CHQ score (0–22)	12	18
Chest radiography	Normal	Normal
24 h Objective cough frequency	6 coughs hr^−1^	16 coughs hr^−1^
Capsaicin cough challenge		
C5	14.9 μmol L^−1^	3.3 μmol L^−1^
CS5	36.2 μmol L^−1^	6.3 μmol L^−1^
Bronchoscopy		
Culture	No growth	No growth
Differential cell count	Alveolar macrophages 95%	Alveolar macrophages 90%
	Neutrophils < 1%	Neutrophils 8%
	Lymphocytes 4–5%	Lymphocytes 2%
	Eosinophils 0%	Eosinophils 0%
Histopathology	Bronchial tissue with no inflammation, including eosinophils	Very mild and non-specific inflammation in the stroma; predominantly lymphocytes and occasional eosinophil
Neurological features		
Duration of neurological symptoms	20 years	1 year
Diagnosis	Advanced CANVAS	Early CANVAS
CANVAS features	All features of CANVAS; autonomic disturbance (orthostatic); cramping pains	Mild peripheral sensation loss (feet) only
Subtypes of pain by NPSI		
Superficial spontaneous burning	0/10	0/10
Deep spontaneous pressing	3/10	0/10
Paroxysmal	10/10	0/10
Evoked	0/10	3/10
Paraesthesia/dysesthesia	0/10	0/10
Walking aids	Stick (1 or 2)	Nil
Arnold’s reflex	Present	Absent
Airway biopsy	Absent pan-neuronal and Aδ epithelial nerve fibres	Severe depletion of pan-neuronal and Aδ epithelial nerve fibres
Unmyelinated total nerve length*	0 (0–0) μm	0 (0–125) μm
A*δ* nerve length*	0 (0–0) μm	0 (0–206) μm
Skin biopsy IENFD (distal leg)	0.0 fibre.mm^−1^	0.0 fibre.mm^−1^
Nerve conduction study	Severe sensory axonal polyneuropathy affecting both upper and lower extremities	Severe sensory axonal polyneuropathy affecting the lower more than upper extremities; may suggest a length-dependent involvement
	Motor responses are normal	Motor responses are normal
Microneurography	Low number of fibres recorded (axon loss). Dysfunction of small nerve fibres, both somatosensory and sympathetic	Lack of C-nociceptors and sympathetic fibres. Suggestive of a loss of somatosensory and sympathetic fibres

*median (IQR). *CANVAS*; Cerebellar ataxia with peripheral neuropathy and vestibular areflexia syndrome; *BMI* body mass index, *VAS* Visual analogue scale, *LCQ* Leicester Cough Questionnaire health status (lower scores indicate worse cough-specific health status), *CHQ* Cough Hypersensitivity Questionnaire (higher scores indicate more cough triggers), *C5* concentration of capsaicin required to evoke five coughs without attempted cough suppression, *CS5* C5 with attempted cough suppression, *IENFD* intraepidermal nerve fibre density (normal range mean ± SD 12.4 ± 4.6 fibre.mm^−1^) [20]; *NPSI,* Neuropathic pain symptom inventory

### Neurological Assessments

#### Assessment of Pain

Pain was assessed during clinical assessment and by the validated neuropathic pain symptom inventory (NPSI) [8]. The NPSI quantifies different neuropathic pain types; higher scores indicate greater symptom burden across pain subtypes.

#### Nerve Conduction Studies

Nerve conduction studies (NCS) were used to assess large-myelinated nerve fibre function. Amplitude and nerve conduction velocity of sensory nerve action potential (SNAP) and compound motor action potential (CMAP) were obtained from upper and lower extremities.

#### Microneurography

Unmyelinated nerve fibre function was assessed using microneurography to record C-fibre action potentials from the superficial peroneal nerve in the foot, using tungsten microelectrodes (200 μm diameter, lacquer-insulated, nominal impedance 1 MΩ). The neural signals were amplified with an isolated, high input impedance amplifier (Neuro Amp Ex, ADInstruments, Australia), bandpass filtered (maximum range 50–5000 Hz), and fed to a noise eliminator (Hum Bug, Quest Scientific, North Vancouver, Canada). Responses to electrical stimulation were digitised with a data acquisition board (National Instruments, PCI-6221, USA) and recorded in a PC using QTRAC software (©Institute of Neurology, London, UK) and displayed as a raster plot of latencies. Latencies of selected units with adequate signal-to-noise were measured from the raw data, so that each dot represented an identified single unit. Specific functional types of unmyelinated peripheral nerve fibres were identified using activity-dependent slowing of conduction velocity using previously described protocols [9].

### RFC1 PCR

Flanking and repeat-primed PCRs were used to identify pathogenic AAGGG and non-pathogenic AAAGG or AAAAG repeat expansions in *RFC1*, as described by Cortese et al. [10]. Positive cases were defined as negative flanking PCR, negative repeat-primed PCR for (AAAGG)_exp_ and (AAAAG)_exp_ configurations, and the presence of a decremental saw-tooth pattern in repeat-primed PCR for the pathogenic AAGGG repeat expansion [10].

### Cough Assessments

#### Patient Reported Outcome Measures

Cough severity was assessed using a 100 mm visual analogue scale (VAS), cough-specific impact with the Leicester Cough Questionnaire (LCQ, range 3–21, lower scores indicating worse impairment), and cough triggers with the Cough Hypersensitivity Questionnaire (CHQ, 0–22, higher scores indicate more triggers) [11–13].

#### Objective Cough Frequency

Cough frequency was assessed over a 24-h period using the validated Leicester Cough Monitor [14], which consists of a wearable digital audio recorder (LFH0662, Philips, Amsterdam, Netherlands), a lapel microphone (LFH9173, Philips, Amsterdam, the Netherlands), and specialised cough detection software [14]. Cough events were recorded as single occurrences, regardless of whether they occurred singly or as part of a bout [14]. The objective cough frequency (coughs.hr^−1^) over 24 h was documented.

#### Inhaled Capsaicin Challenge Test

Cough reflex sensitivity was evaluated with inhaled capsaicin cough challenge test following the recommendations of the European Respiratory Society [15]. Patients had not experienced an upper respiratory tract infection within the previous 6 weeks. Diluted capsaicin was administered via single-breath inhalations of increasing doubling doses (0.49–1000 μmol.L^−1^) using an air-powered digital dosimeter (KoKo Digidoser; nSpire Health Inc., Longmont, CO, USA) at 1-min intervals. Potential anticipation effects were mitigated by interspersing randomly administered inhalations of 0.9% saline solution [15]. A consistent nebuliser (Model 646; DeVilbiss Healthcare, Port Washington, NY, USA) with an output of 1.205 mL·min^−1^ was utilised for all patients. Inspiratory flow was maintained at 0.5L·s^−1^ using a valve [15]. A minimum of three respiratory cycles occurred before the nebulised solution was administered, with concurrent monitoring of flow-volume signals to ensure consistent and maximal inspiratory effort (0.5 L s^−1^) throughout the administration. The test was repeated if sub-maximal inhalation was observed. Following administration, coughs were counted for a 15-s period with the assistance of an MP3 recorder (LFH0662, Philips, Amsterdam, Netherlands) [15]. The capsaicin challenge test was concluded once ≥ 5 coughs were elicited by a single dose administration. The capsaicin concentrations required to elicit two (C2) and five (C5) coughs were determined through interpolation, with lower levels indicating greater cough reflex sensitivity [16]. Normal ranges for C2 and C5, utilising the same methodology and equipment as in this study, have been previously reported for men and women [17]: C2 range: 4.44–18.2 μmol L^−1^ and 7.63–35.9 μmol L^−1^, and C5 range: > 37.8 μmol L^−1^ and > 158 μmol L^−1^, respectively. More than 48 h after testing of cough reflex sensitivity, the test was repeated with the patient instructed to “not cough during the investigation”. The suppressed C2 (CS2) and C5 (CS5) were again calculated by interpolation [18].

#### Spirometry

Spirometry (Jaeger MS-PFT Analyser Unit with Sentry Suite software version 2.19.96) was measured according to guidelines by the European Respiratory Society guidelines and the American Thoracic Society [19]. FeNO was measured with the NIOX VERO analyser (Aerocrine AB, Solna, Sweden).

### Bronchoscopy, Bronchoalveolar Lavage, and Bronchial Biopsies

Fibreoptic bronchoscopy was performed under conscious sedation (fentanyl and midazolam) with topical lidocaine as per local guidelines. Fluid from bronchoalveolar lavage was sent for differential cell count. 4–6 endobronchial forceps biopsies were obtained from the bronchus intermedius.

### Histologic Assessments

Airway biopsies (~ 1–2 mm^3^) were obtained from the bronchus intermedius using fibreoptic bronchoscopy or excised from deceased donor airways and were formalin fixed and immunostained as previously described [6]. Briefly, samples were washed with tris-buffered saline (TBS) and blocked overnight at 4 °C with a solution of 1% Triton X-100, 4% normal goat serum, and 5% powdered milk in TBS. Samples were labelled with antibodies against protein gene product 9.5 (PGP9.5, a pan-axonal marker; Millipore) and neurofilament heavy chain (mechanosensitive Aδ sensory axons; Abcam), followed by secondary anti-rabbit 488 and anti-chicken 647 antibodies (Life Technologies), and then counterstained for nuclei with DAPI. Tissues were mounted on well slides (1-mm thick), covered with a glass coverslip, and sealed with Permount (Thermo Fisher Scientific).

Two to three non-overlapping three-dimensional image Z stacks were obtained from each whole-mount sample using an LSM980 confocal microscope (63X, 1.2 NA). Two samples were analysed per study subject. From these image Z stacks, total axonal length and Aδ-positive nerve length were quantified by applying a computer-generated nerve model over PGP9.5-positive voxels and neurofilament-positive voxels, respectively (Imaris) [6].

For dermal biopsies, intraepidermal nerve fibre density (IENFD) was assessed by immunostaining for PGP9.5 in 50 µm tissue Sects. (3 mm punch skin biopsies; distal leg) following standard methods [20].

### Statistical Analysis

All data are expressed as median (interquartile range, IQR), except capsaicin threshold concentrations and cough frequency which were presented as geometric mean (geometric standard deviation, SD).

## Results

### Patient Characteristics

In the father and son (aged 62 and 37 respectively), the duration of cough was 37 and 9 years, and the duration of other neurological symptoms was 20 and 1 years, respectively. The father had advanced CANVAS, displaying all characteristic features, and used walking aids (Table 1). The son had early CANVAS, with mild sensory neuropathy only.

### Neurological Assessments

**Fig. 1:**
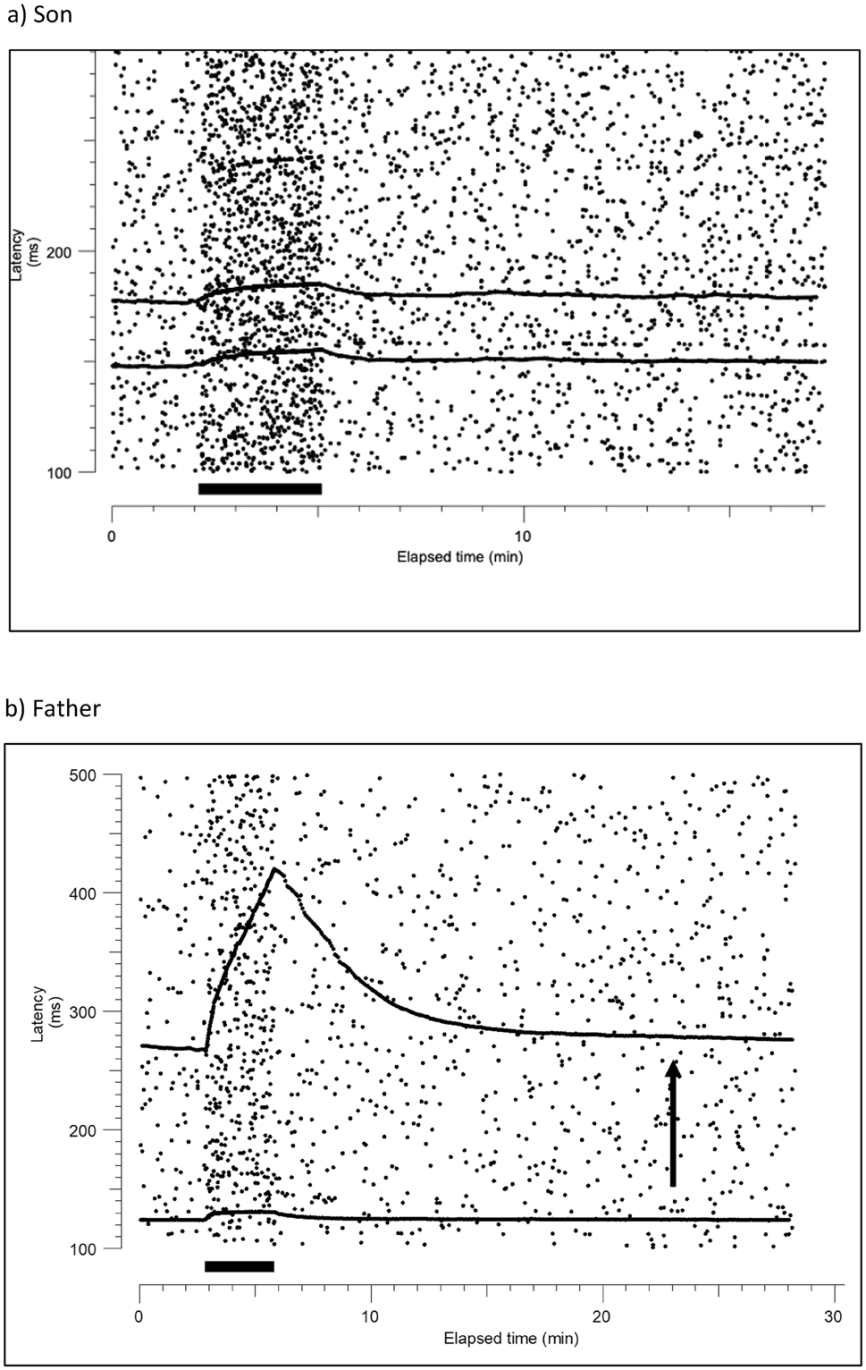
Microneurography cutaneous recordings in CANVAS. A tungsten microelectrode was used to record C-fibre action potentials from the superficial peroneal nerve at the dorsum of the foot from both patients. Activity-dependent slowing of conduction velocity upon repetitive stimulation produces characteristic profiles of latency increase during a 3-min 2 Hz stimulation period (filled bars) interrupting a baseline constant stimulation at 0.25 Hz. The response to stimulation of nociceptors, but not to thermoreceptors, was recorded (arrow), **a **Son: raster plot showing only two C-fibres, identified as thermoreceptors, are seen at latencies ~ 150 and 180 ms. No nociceptors were identified. **b** Father: two C-fibres (one thermoreceptor, one nociceptor) are seen at latencies ~ 120 and 270 ms. The fibre at 270 ms has a profile identifying it as a mechanosensitive C-nociceptor. However, this unit did not respond to repeated application of 512 mN mechanical probe (arrow), suggesting a loss of mechanical transduction or a dying back retraction of the skin nerve terminals

Nerve conduction studies were compatible with a severe, purely axonal, symmetrical sensory neuropathy with normal motor studies in both patients (Table 1). NPSI scores revealed mild deep spontaneous pressing pain (3/10) and severe paroxysmal pain (10/10) in the father, and mild evoked pain (3/10) only in the son. Microneurography revealed an absence of recordable sensory C-nociceptors in the son and a severe reduction in the father, without evidence of spontaneous activity. These findings may reflect a severe loss of somatosensory axons from the superficial peroneal nerve (Fig. 1).

### Cough Assessments

The father and son reported moderately raised cough severity (VAS 58 and 54) and objective cough frequency (6 coughs hr^−1^ and 16 coughs hr^−1^) compared to historical healthy controls (geometric mean [SD] 2 [1] coughs hr^−1^) [21], respectively (Table 1). Cough-specific health status LCQ total score was impaired in both the father (15.9) and the son (13.1). Multiple laryngeal sensations and triggers were reported in the father and son; CHQ total score 12 and 18, respectively. The capsaicin concentration to evoke 5 coughs (C5) was 14.9 μmol L^−1^ in the father and 3.3 μmol L^−1^ in the son, indicating heightened cough reflex sensitivity compared to historical healthy controls (geometric mean [SD] C5 159 [0.6] mmol L^−1^) [16], and comparable to previous RCC and CANVAS data [3]. The concentration of capsaicin causing five coughs whilst attempting cough suppression (CS5) in the father and son was 36.2 and 6.3 μmol.L^−1^, respectively, suggesting partial suppressibility of cough compared to healthy controls data from a previous study [22]. The father reported an urge-to-cough sensation in his throat following inhalation of capsaicin, but in the son it was difficult to discern urge-to-cough sensation as he coughed at very low capsaicin thresholds. Fibreoptic bronchoscopy was grossly normal in the father and son, and differential cell counts from bronchoalveolar lavage were within normal ranges (Table 1).

### Histological Assessments: Airway and Skin

**Fig. 2:**
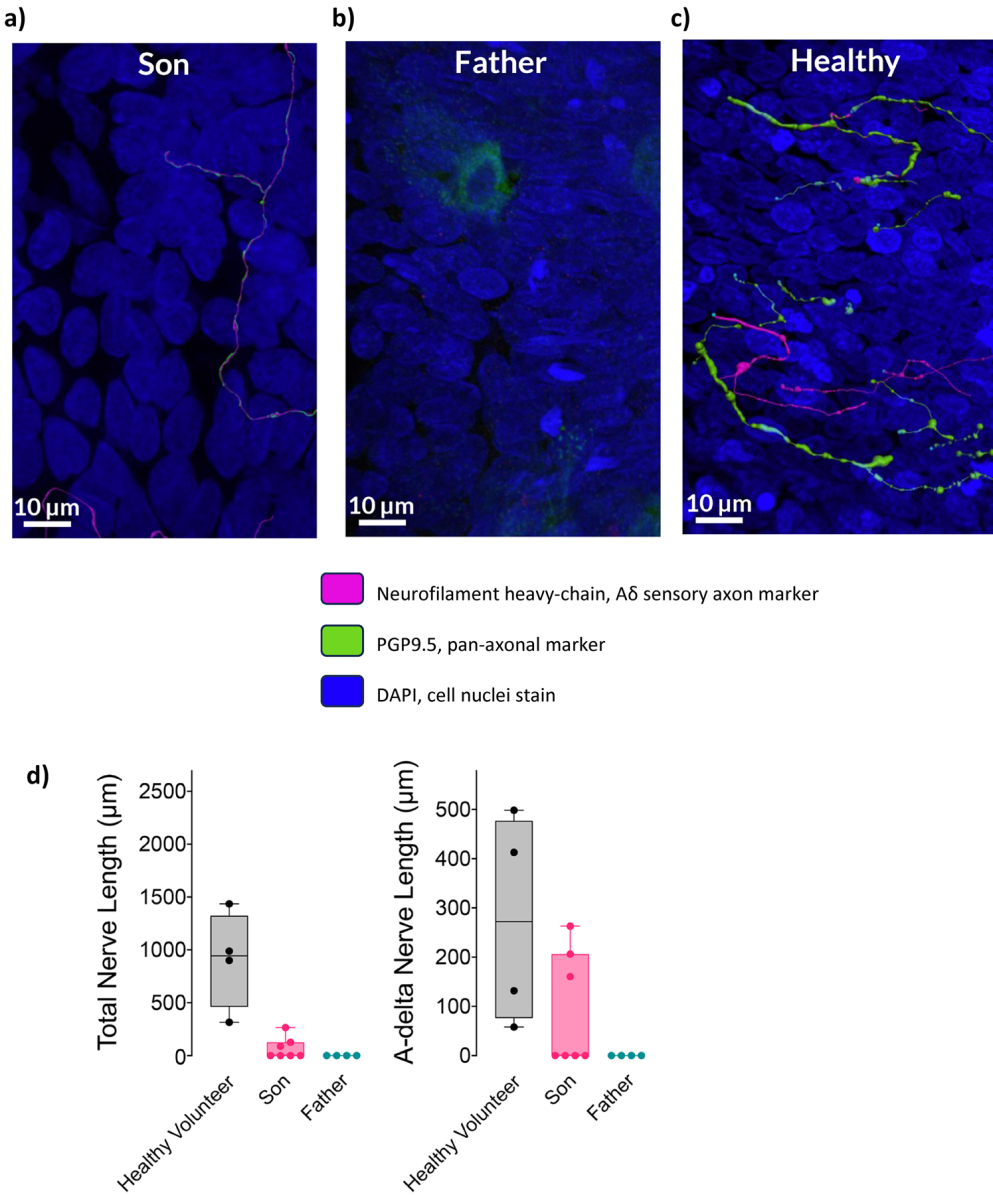
Airway epithelial innervation is severely reduced in patients with CANVAS. Representative images from bronchoscopic biopsies showing near total loss of airway innervation within epithelium in a father (**a**) and son (**b**) with CANVAS as compared to an unrelated airway donor with healthy lungs (**c**). Nerves were immunostained using the pan-axonal marker PGP9.5 (green) and the mechanosensitive A*δ* sensory fibre marker neurofilament heavy chain (pink). Three-dimensional nerve models were generated based on immunopositive voxels (LSM980 63X). **d** Total airway epithelial nerve length and A*δ* sensory nerve length were quantified from nerve models within non-overlapping image Z stacks. Each data point represents nerve length within a non-overlapping image Z stack. Box plots indicated medians, and 25th and 75th percentiles. Error bars indicate min and max

**Fig. 3:**
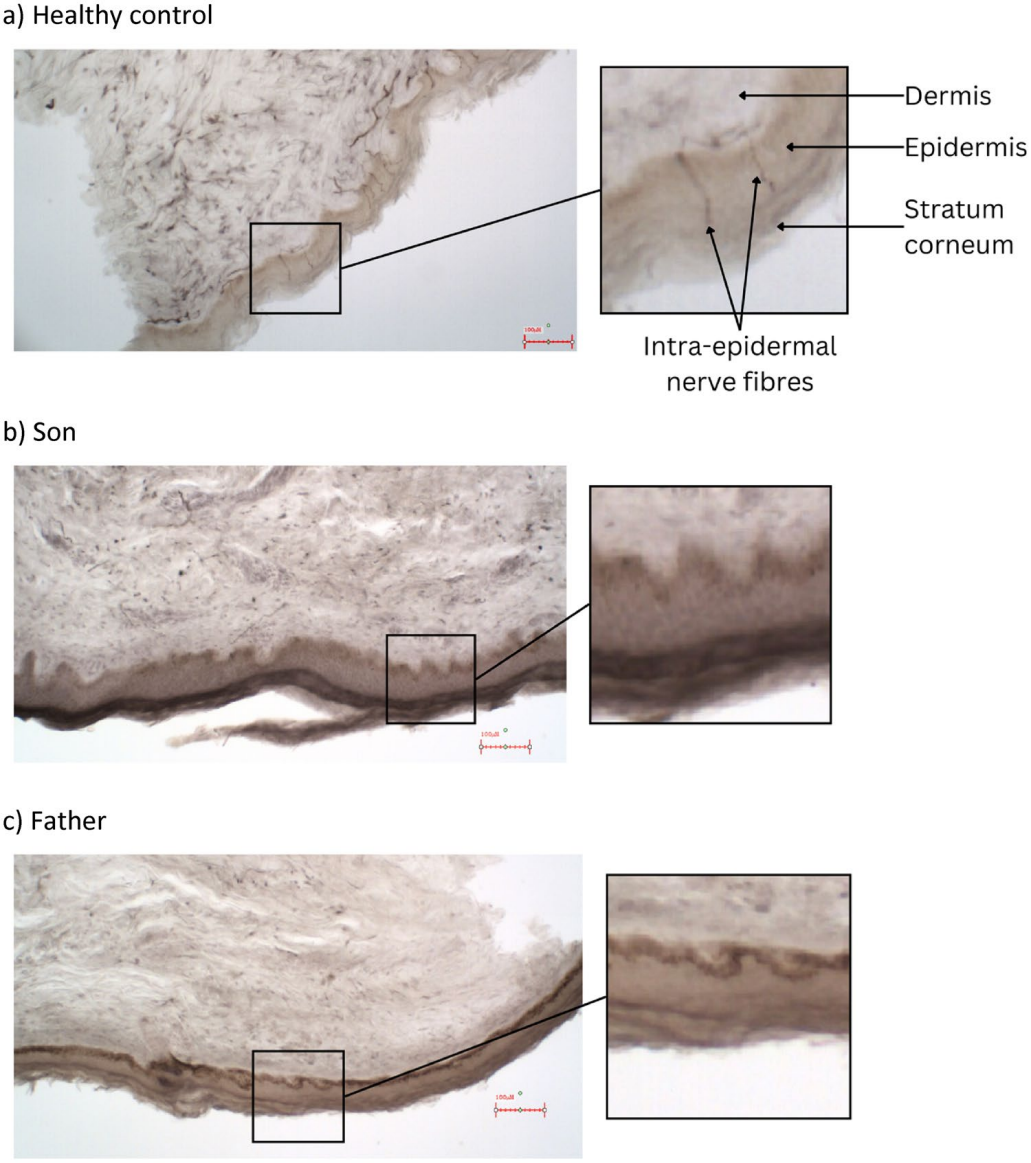
Skin biopsy images from the ankle of a healthy control, the son, and the father: Skin biopsies were evaluated using pan-neuronal PGP9.5 bright-field immunohistochemistry to assess intraepidermal nerve fibre density (IENFD). No intraepidermal small fibres were observed in either the father or son, although nerve fibres were present in the dermis. Images show **a** a healthy control, **b** the son, and **c** the father. In both (**b**) and (**c**), deep dermal fibres are clearly visible but fail to penetrate into the epidermis. IENFD was 0.0 fibres mm^−1^ for both the son and father, compared to 8.4 fibres.mm^−1^ for the healthy control

Histopathological examination of bronchial biopsies revealed normal stroma and cells in both patients (Table 1). However, PGP9.5-positive unmyelinated nerve fibres were absent in the father and severely reduced in the son compared to the deceased donor healthy control: median (IQR) total nerve fibre length 0 (0–0) μm, 0 (0–125) μm, and 944 (461–1323) μm, respectively (Table 1, Fig. 2). Neurofilament-positive Aδ fibres were also absent in the father and reduced in the son compared to the healthy control: median (IQR) Aδ nerve fibre length 0 (0–0) μm, 0 (0–206) μm, and 272 (76–477) μm, respectively. Skin biopsies demonstrated absent intraepidermal nerve fibres in both patients (Fig. 3). Intraepidermal nerve fibre density (IENFD) was 0.0 fibres mm^−1^ for both the son and father, compared to 8.4 fibres mm^−1^ for the healthy control.

## Discussion

This is the first report of absence or severe depletion of bronchial airway nerve endings in CANVAS patients with chronic cough. Despite this, both patients had cough reflex hypersensitivity to capsaicin. As expected, both patients had absence of intraepidermal nerve fibres in skin biopsies, and impaired myelinated and unmyelinated sensory nerve function in the limbs. Bronchial nerve depletion was more severe in the father than the son, consistent with the progressive loss and destruction of neurons over time in CANVAS [1].

The mechanism of cough and increased airway sensitivity in patients with CANVAS is unknown. A possible mechanism is denervation hypersensitivity. CANVAS is a ganglionopathy, involving neuron cell death. Denervation hypersensitivity is a centrally mediated phenomenon that occurs when there is a reduction in peripheral inputs (e.g. from airway afferents) to secondary (central) neurons [23]. Thus, even a small number of remaining viable peripheral airway nerves may generate very large responses (such as cough or pain hypersensitivity) because the central networks that receive this input are now primed to be hypersensitive [23–25]. This phenomenon has been described in other neuropathic pain conditions such as postherpetic and diabetic neuralgia [23, 26], supported by evidence from functional MRI (fMRI) studies [23–25, 27, 28]. Moreover, centrally mediated cough hypersensitivity is considered a key mechanism in RCC. fMRI studies have demonstrated increased activation of cough centres, decreased activation of cough suppression centres, and abnormal midbrain amplification of afferent inputs in patients with RCC compared to controls [29, 30]. Similar mechanisms may be relevant in CANVAS cough.

In the early stages, the cough in CANVAS may result from dysfunctional nerves, nerves in early stages of cell death, or possibly nerve sprouting. Cough and hypersensitivity in CANVAS may be analogous to spontaneous pain and increased peripheral sensitivity in neuropathic pain. This concept is based on common mechanisms of sensory dysregulation, where altered nerve function leads to hypersensitivity and spontaneous symptoms [24, 31]. Patients with CANVAS can experience episodes of chronic cough that occur spontaneously without any obvious external triggers [32]. This could be due to abnormal, spontaneous firing of sensory nerves in the airways (possibly vagal afferents), akin to damaged or dysfunctional nerves in neuropathic pain [24]. Spontaneous sensory nerve activity may cause cough hyperresponsiveness [31], analogous to allodynia (a noxious perception generated by a non-noxious stimulus) or hyperalgesia (increased sensitivity to pain) seen in neuropathic pain conditions [33]. Over time, repeated cough reflex activation may lead to central sensitisation, where the central nervous system amplifies responses to stimuli, as in chronic pain conditions [24]. Maladaptive nerve sprouting after neuronal injury has been reported in hypersensitive neuropathic pain [34] and may be a possible mechanism of cough in CANVAS. Electron microscopy studies to investigate sprouting in viable neurons are needed in CANVAS.

Aside from being a small case series, our report has limitations. Comparison of airway innervation was made with a single healthy subject; however, this was in keeping with previously reported airway nerve length in a larger series of healthy subjects [6]. Consistent with previous airway innervation studies [6], we sampled epithelium only from the large airways. Future research should sample alternative airway sites, such as the larynx, to confirm nerve depletion is present throughout. The airway nerve density in our patients and healthy subjects may have been impacted by natural sample variation. In animal models, biopsies from parts of the airway can be devoid of innervation [35–37]. In humans, airway innervation varies between subjects and within subjects depending on the biopsy location [6]. We have attempted to minimise these potential confounders by standardising the airway location assessed, assessing more than one biopsy for each subject and measuring multiple z stacks from each sample. Furthermore, it is possible that expression of PGP9.5 may be lost in the diseased neurons in CANVAS, such as reported in conventional small fibre neuropathy [20], and hence some nerve fibres may not have been visible with our methodology. A further limitation is that the function of the remaining C-fibres in our patient was not investigated as it is difficult to study in patients. There are subtypes of airway C-fibres that may activate or inhibit cough [38–40]. The role of airway nerve fibres in activating or inhibiting cough in CANVAS needs further study. The cough reflex is initiated when vagal afferents arising from the airway epithelium are triggered by stimuli such as capsaicin. However, capsaicin can trigger the release of local inflammatory cytokines, such as IL-6 [41]. These may sensitise nerve fibres deeper than the epithelium, thereby causing cough. Alternative mechanisms of peripheral cough reflex hypersensitivity therefore need consideration.

In summary, CANVAS-associated cough might be described as a "neuropathic cough", whereby sensory nerves in the airways behave abnormally due to underlying neuropathy, and might benefit from therapies targeting neural dysregulation. The underlying mechanisms may evolve with the duration of disease, with earlier predominance of peripheral sensitisation, and later central sensitisation. It will be important to develop better clinical tools to distinguish these two main mechanisms and ideally tailored treatments for each.

## Data Availability

The authors confirm that the data supporting the findings of this study are available within the article.
